# Supplementary material for: Cancer Essential Genes Stratified Lung Adenocarcinoma Patients with Distinct Survival Outcomes and Identified a Subgroup from the Terminal Respiratory Unit Type with Different Proliferative Signatures in Multiple Cohorts
Source: Cancers (Basel). 2021 Apr 28;13(9):2128. doi: 10.3390/cancers13092128 (PMC8124388; doi:10.3390/cancers13092128)
Supplement: Supplementary file 1 [file cancers-13-02128-s001.zip › cancers-1183152-supplementary/cancers- 1183152 supplementary.pdf]

cancers

Supplementary Materials

Cancer Essential Genes Stratified Lung Adenocarcinoma Patients with Distinct Survival Outcomes and Identified a Subgroup from the Terminal Respiratory Unit Type with Different Proliferative Signatures in Multiple Cohorts

Kuo-Hao Ho [1,2], Tzu-Wen Huang [1,3], Ann-Jeng Liu [4], Chwen-Ming Shih [1,2,*] and Ku-Chung Chen [1,2,*]

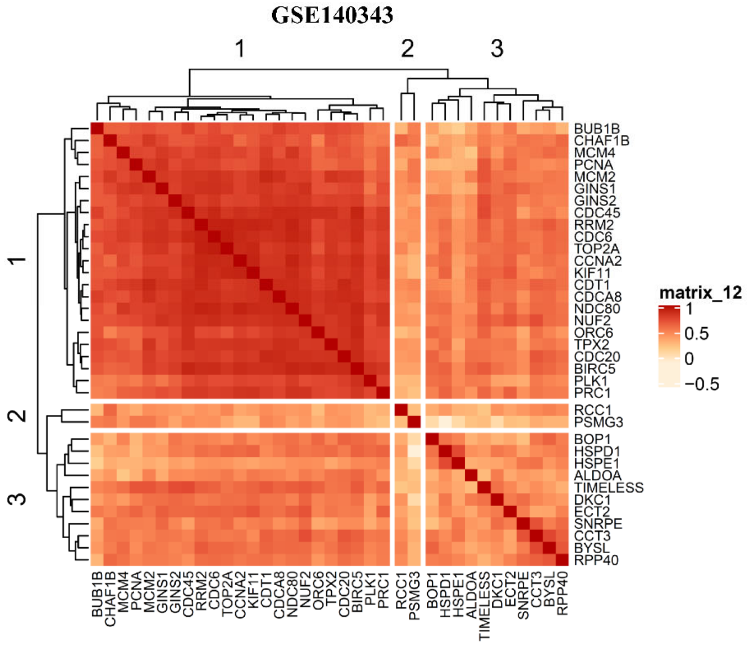

Figure S1. Associations of cancer essential genes from GSE140343 data shown as a correlation heatmap.

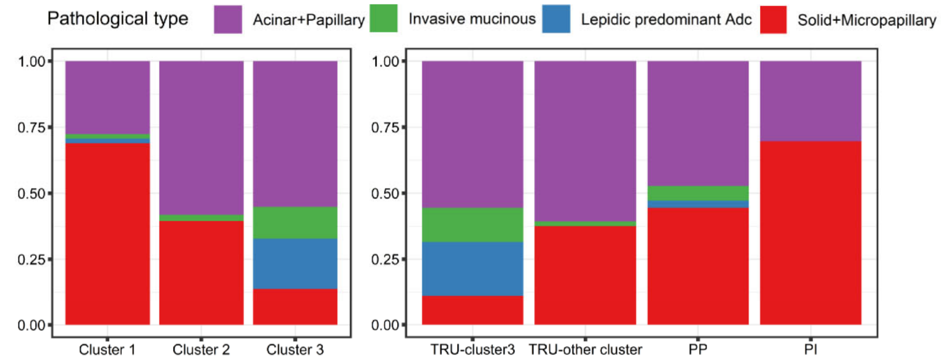

Fisher exact test p value <0.01, odd ratio = 5.35		
	Solid + Micropapillary	Other pathological types
Cluster 1	40	18
Other clusters	42	102

Fisher exact test p value <0.01, odd ratio = 32.86		
	Lepidic	Other pathological types
Cluster 3	11	47
Other clusters	1	143

Fisher exact test p value =0.02, odd ratio = 1.96		
	Acinar + Papillary	Other pathological types
Cluster 2	50	36
Other clusters	48	68

Fisher exact test p value <0.01, odd ratio = 36.86		
	Lepidic	Other pathological types
TRU-Cluster 3	11	43
Other types	1	147

Figure S2. Association between pathological type and essential gene-stratified cluster in LUAD patients. The ratios of LUAD patients with different pathological types were presented in stacked bar plot. The 2x2 contingency tables which indicated the number of patients in a given cluster and pathological type were shown. A Fisher exact test was conducted to investigate their association.

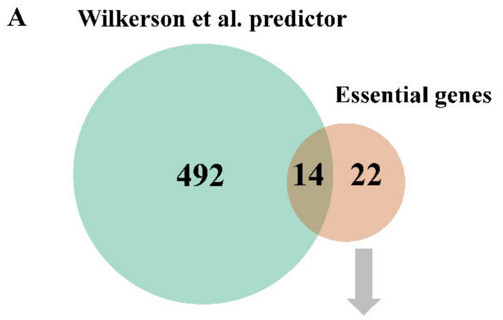

Twenty-two selected essential genes

ALDOA	MCM2	CDCA8
BYSL	CDC6	ORC6
MCM4	GINS2	PSMG3
CHAF1B	NDC80	BIRC5
DKC1	RPP40	CDC45
BOP1	PLK1	SNRPE
TIMELESS	CDT1	
RCC1	NUF2	

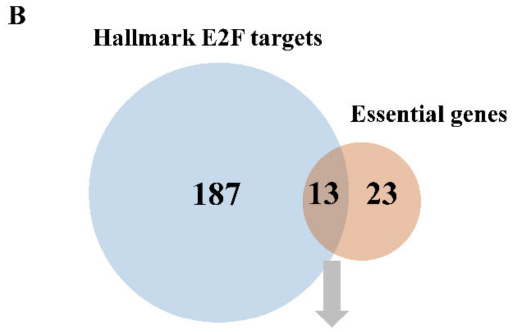

BIRC5	PCNA
BUB1B	PLK1
CDC20	RRM2
CDCA8	TIMELESS
GINS1	TOP2A
MCM2	ORC6
MCM4	

Figure S3. List of 22 selected essential genes and 13 E2F target genes. (A) selected essential genes (B) E2F target genes.

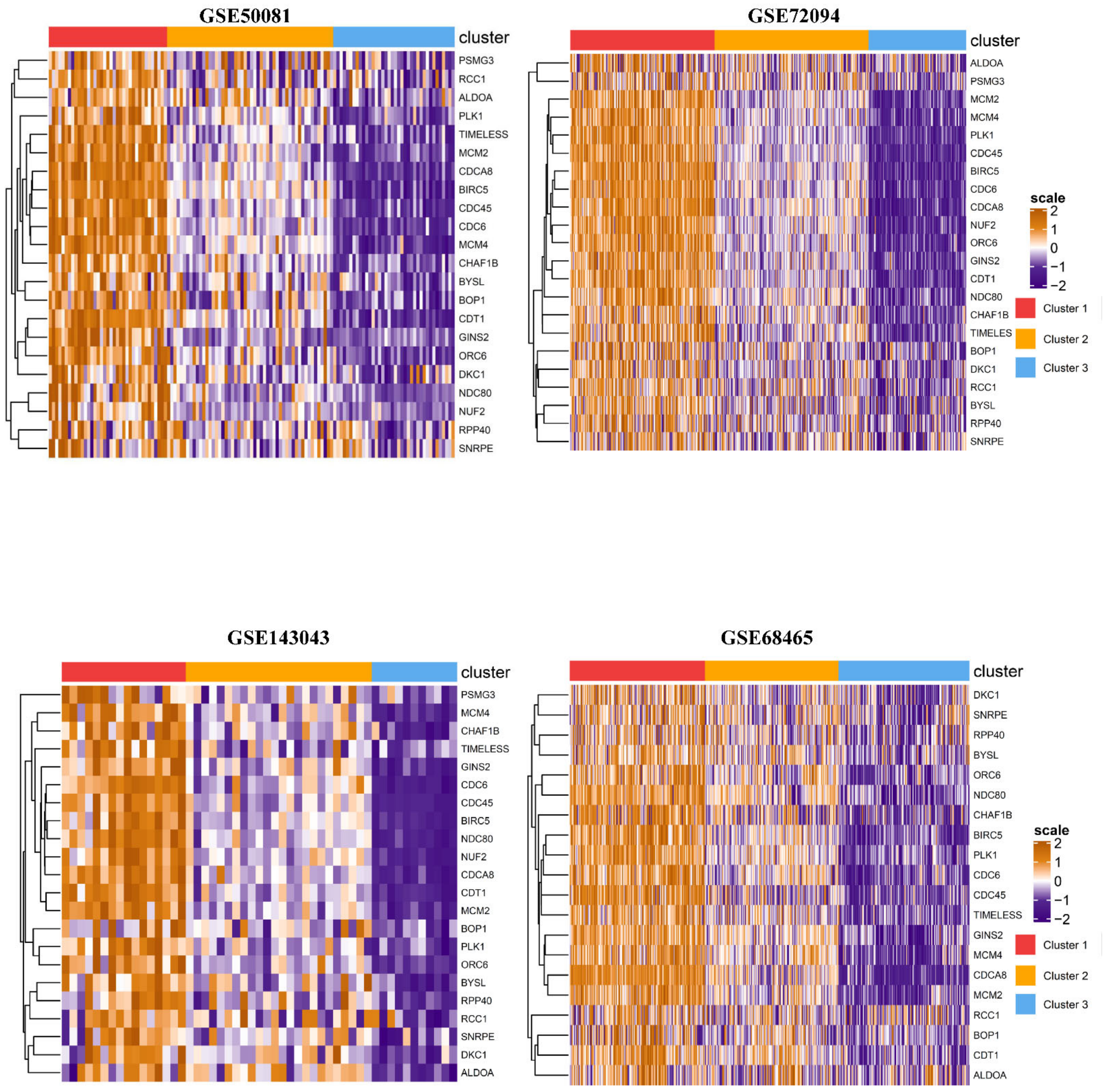

GSE50081
cluster
PSMG3
RCC1
ALDOA
PLK1
TIMELESS
MCM2
CDCA8
BIRC5
CDC45
CDC6
MCM4
CHAF1B
BYSL
BOP1
CDT1
GINS2
ORC6
DKC1
NDC80
NUF2
RPP40
SNRPE
GSE72094
cluster
ALDOA
PSMG3
MCM2
MCM4
PLK1
CDC45
BIRC5
CDC6
CDCA8
NUF2
ORC6
GINS2
CDT1
NDC80
CHAF1B
TIMELES
BOP1
DKC1
RCC1
BYSL
RPP40
SNRPE
scale
2
1
0
−1
−2
Cluster 1
Cluster 2
Cluster 3
GSE143043
cluster
PSMG3
MCM4
CHAF1B
TIMELESS
GINS2
CDC6
CDC45
BIRC5
NDC80
NUF2
CDCA8
CDT1
MCM2
BOP1
PLK1
ORC6
BYSL
RPP40
RCC1
SNRPE
DKC1
ALDOA
GSE68465
cluster
DKC1
SNRPE
RPP40
BYSL
ORC6
NDC80
CHAF1B
BIRC5
PLK1
CDC6
CDC45
TIMELESS
GINS2
MCM4
CDCA8
MCM2
RCC1
BOP1
CDT1
ALDOA
scale
2
1
0
−1
−2
Cluster 1
Cluster 2
Cluster 3

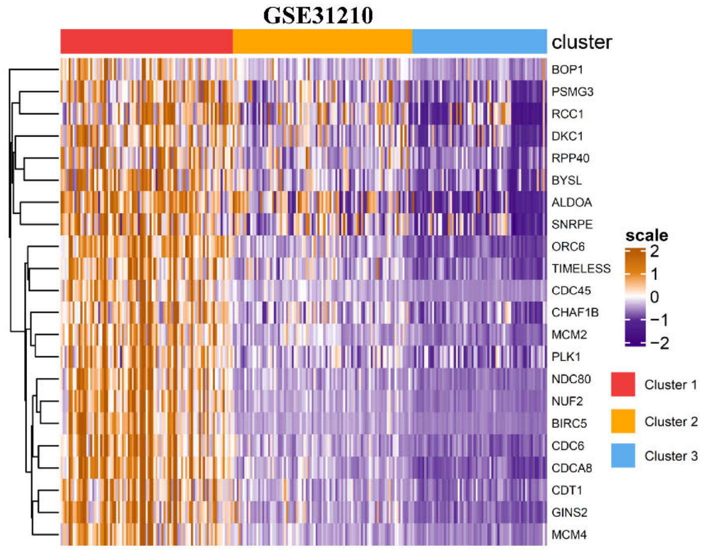

Figure S4. Validation of expression patterns in essential gene-classified clusters from GEO datasets. A heatmap demonstrates patterns of essential gene expressions in three clusters from the GSE140343, GSE68465, GSE72094, GSE50081, and GSE31210 datasets.

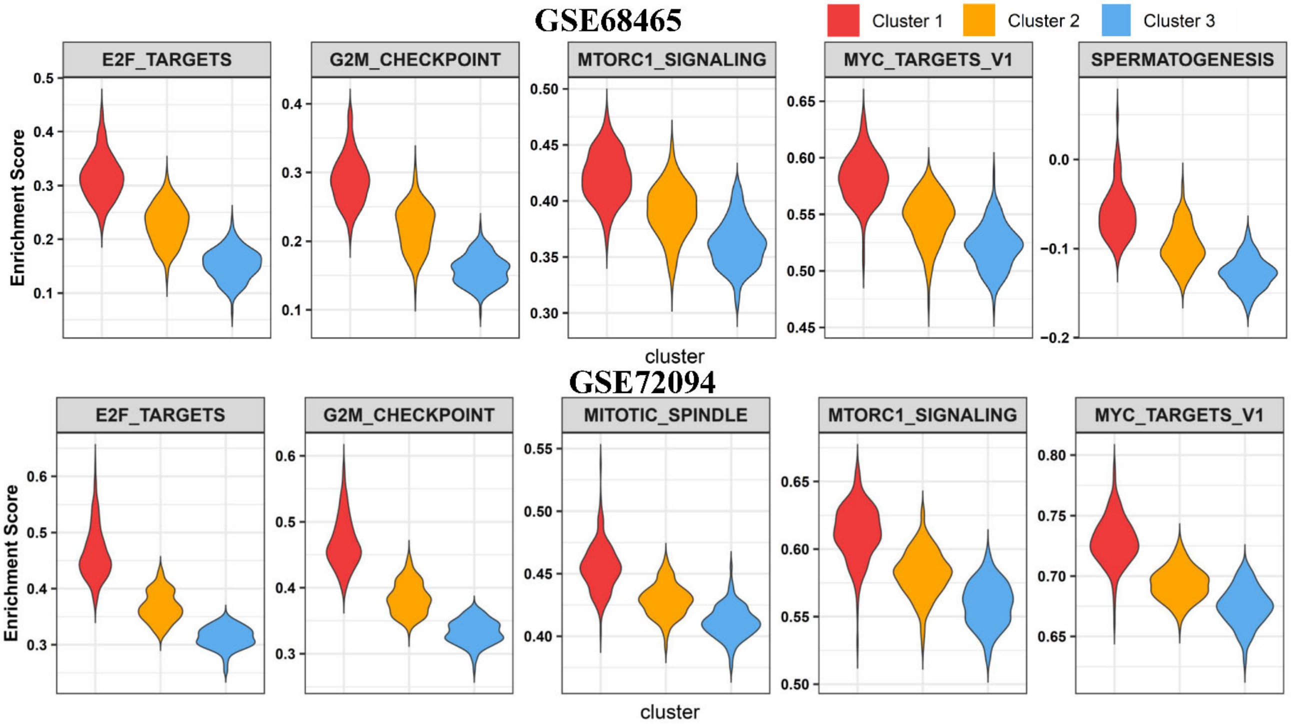

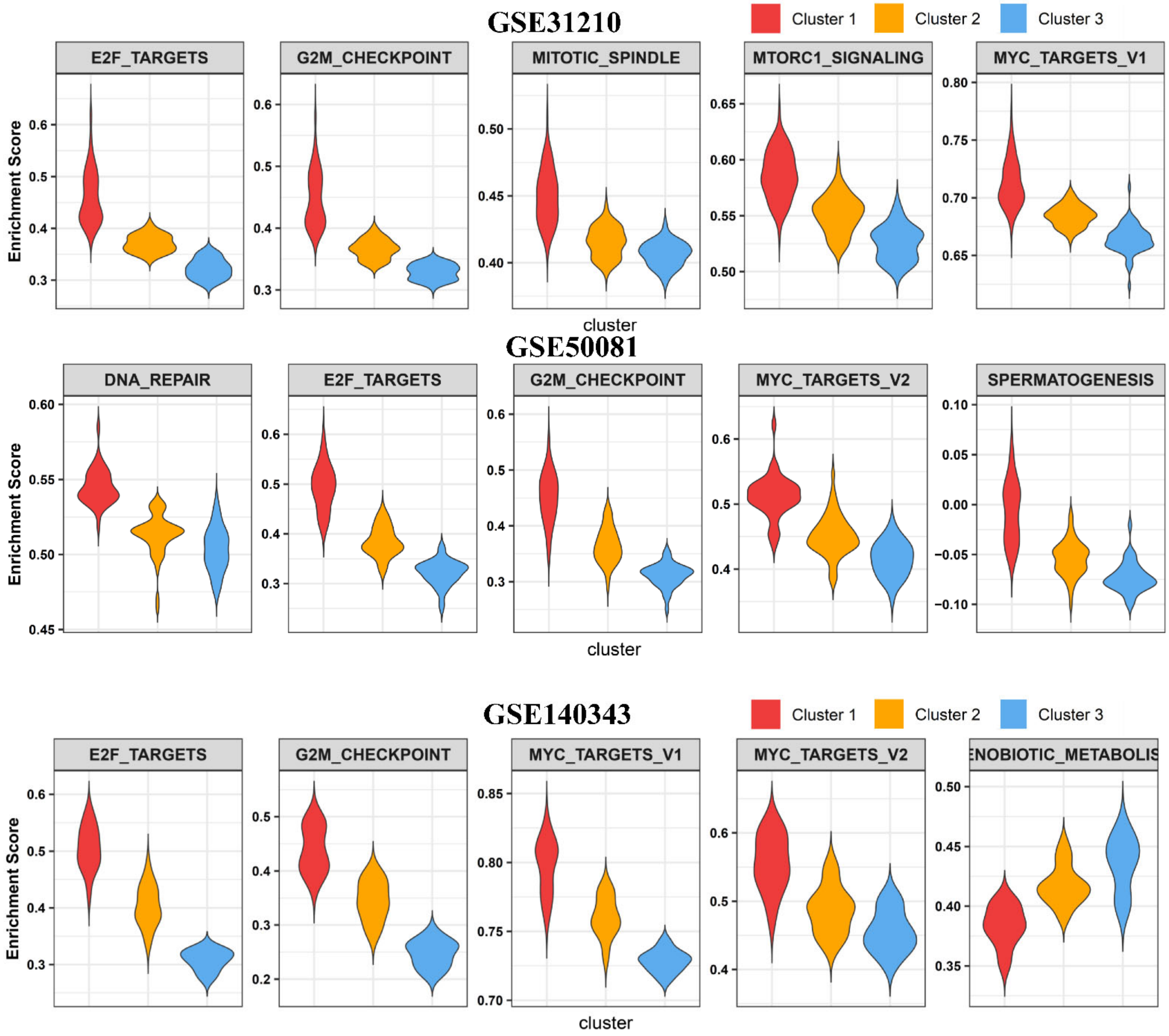

Figure S5. Validation of associated signaling in essential gene-classified clusters from GEO datasets. ssGSEA scores of top signaling pathways from the GSE140343, GSE68465, GSE72094, GSE50081, and GSE31210 datasets are shown as violin plots.

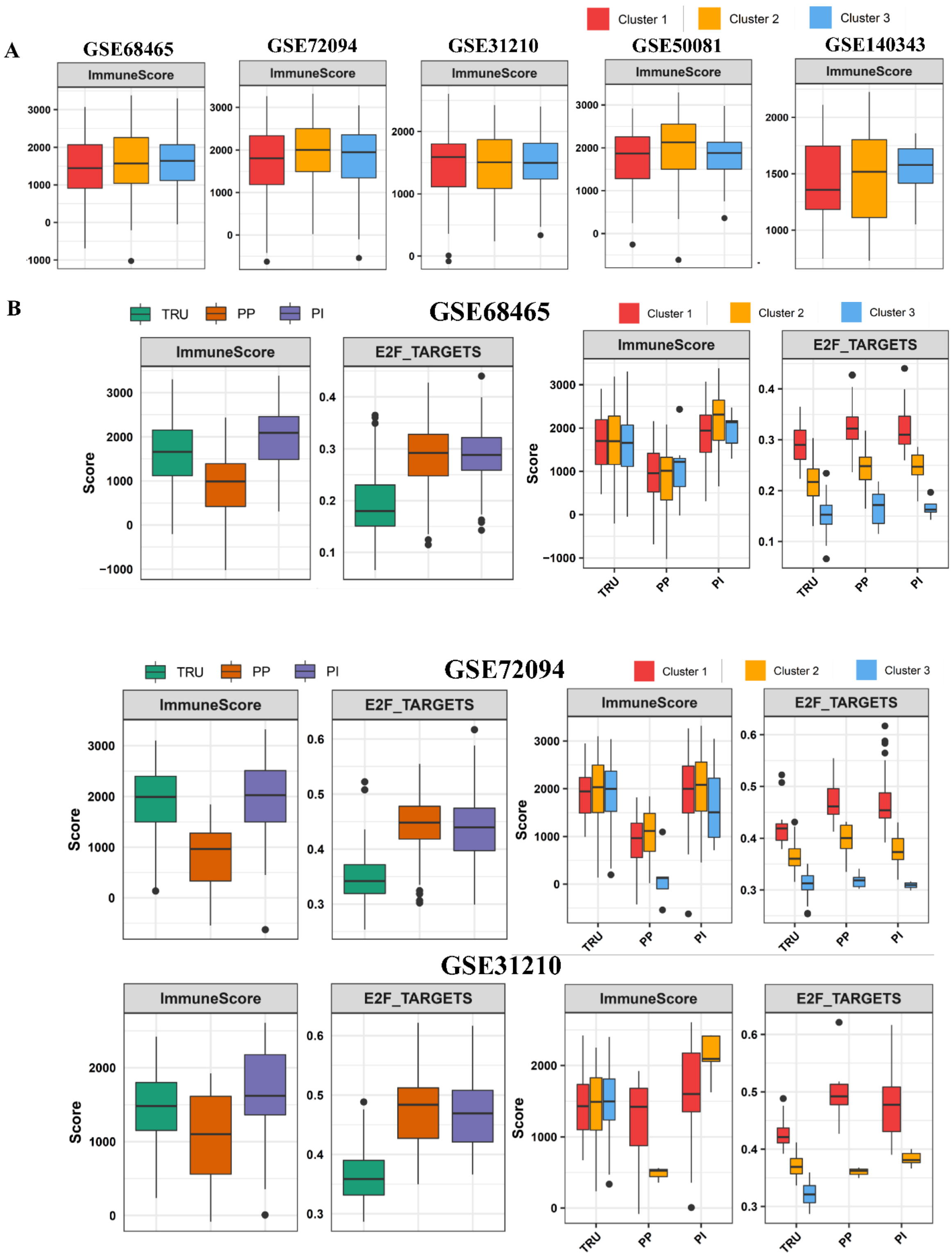
A
GSE68465
GSE72094
GSE31210
GSE50081
GSE140343
ImmuneScore
Cluster 1
Cluster 2
Cluster 3
B
TRU
PP
PI
E2F_TARGETS
Score

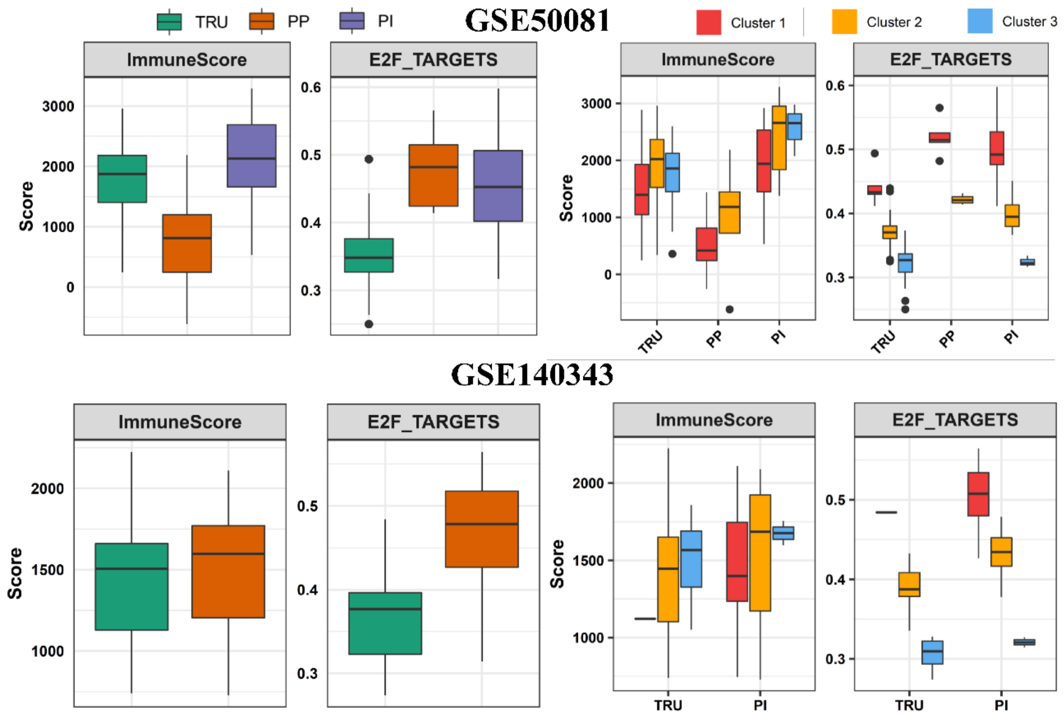

Figure S6. Immune infiltration and E2F signaling profiles of lung adenocarcinoma (LUAD) patients in GEO datasets. (A) ESTIMATE-derived immune infiltration levels from the GSE140343, GSE68465, GSE72094, GSE50081, and GSE31210 datasets were compared among essential gene-stratified clusters and are shown as boxplots. (B) ESTIMATE-derived immune infiltration levels and ssGSEA-inferred E2F target signaling from the GSE140343, GSE68465, GSE72094, GSE50081, and GSE31210 datasets were compared among previously reported expression subtypes with or without subdivision into essential gene-classified clusters.
